# When epilepsy triggers psychosis : understanding the mystery of postictal psychosis

**DOI:** 10.1192/j.eurpsy.2025.895

**Published:** 2025-08-26

**Authors:** A. Tarrada

**Affiliations:** 1 Neuropsychiatry Unit, UMC Central, Nancy; 2 Neuropsychiatry Unit, Centre Psychothérapique de Nancy, Laxou, France

## Abstract

**Introduction:**

Postictal Psychosis (PIP) is a severe psychiatric complication of epilepsy, defined by a brief psychotic episode, with a mean duration about 10 days, occurring after a cluster of seizures, or a focal seizure evolving to bilateral tonic-clonic seizure, and sometimes after a single seizure. Around 2 **%** of patients are affected by this disorder, mostly in long-lasting, drug-resistant temporal lobe epilepsy, but its prevalence is probably underestimated. (Kanemoto et al. Cambridge, 2011; p.67-79). PIP can lead to severe behavioral disturbances, such as agitation, violence and suicide (Tarrada et al. Epilepsy Behav, 2022). Multiple hypotheses exist about the pathophysiology of Postictal Psychosis (de Toffol et al. Encephale, 2016; p443-447). As the clinical manifestations of PIP are roughly stereotyped regardless of the epileptogenic zone, we assumed the existence of a common neurological pathway.

**Objectives:**

We aimed to determine if a specific brain network sustained the psychotic episode, regardless of the localization of the epileptogenic zone.

**Methods:**

We conducted a systematic review following the PRISMA guidelines (Cheval et al. Seizures, 2024; p 44-55). We included studies that provided EEG, and metabolic imaging performed during the presence of psychotic symptoms. Studies were not included, when the diagnosis of PIP was doubtful, or when the results EEG and/or imaging were not detailed enough.

**Results:**

We included a total of 24 studies providing electrophysiological results(n=22) and metabolic imaging performed during the PIP(n=5). Temporal and frontal lobes seemed frequently involved, without clear evidence for lateralization. The EEG patterns were heterogenous, varying from unchanged to diffuse slowing. Metabolic pattern showed an increased perfusion within temporal and frontal lobes during PIP. These results correspond to the patterns described during postictal state, but they persisted throughout PIP, within regions larger than the epileptogenic zone and resolved with the recovery (Cheval et al. Seizures, 2024; p 44-55).

**Conclusions:**

PIP symptoms are associated with an excessive persistence of postictal changes within extended frontotemporal networks. A hypothesis could be that PIP results from an abnormally prolonged and diffuse post-ictal dysregulation. Nonetheless this results is relatively imprecise and further studies should elucidate the subjective experience of the epileptic seizure, and the neuropsychological profile of patients with PIP episodes. Finally, these results could be also compared to the results of invasive EEG in patients with schizophrenia obtained in a previous controversial study, that showed the presence of epileptic activities in depth regions of the brain (Heath et al. Epilepsy Behav, 2005; p633-645), a pattern distinct from PIP, that would suggest epileptic psychoses have a pathophysiology different from primary psychoses.

**Disclosure of Interest:**

None Declared

